# Maedi-visna infection in Pomeranian Coarsewool Sheep in Germany: seroprevalence, environmental and genetic risk factors

**DOI:** 10.3389/fvets.2025.1708380

**Published:** 2025-11-27

**Authors:** Cassandra Frölich, Maria Serena Beato, Paola Gobbi, Sara Mrabet, Gesine Lühken, Martin Ganter

**Affiliations:** 1 Clinic for Swine and Small Ruminants, University of Veterinary Medicine Hannover, Foundation, Hannover, Germany; 2 National Reference Laboratory for Ruminant Retroviruses (CEREL), Istituto Zooprofilattico Sperimentale dell’Umbria e delle Marche “Togo Rosati” (IZSUM), Perugia, Italy; 3 Department of Animal Breeding and Genetics, Justus Liebig University of Gießen, Gießen, Germany

**Keywords:** small ruminant lentivirus, maedi-visna virus, Pomeranian Coarsewool Sheep, seroprevalence, risk factor, *TMEM154*, ELISA, Germany

## Abstract

Small ruminant lentiviruses (SRLV) cause maedi-visna (MV), an incurable, wasting disease, affecting sheep worldwide. This study evaluated the seroprevalence of maedi-visna virus (MVV) in the native German sheep breed Pomeranian Coarsewool Sheep (RPL) and environmental and genetic risk factors for individual and flock-level seropositivity. A total of 849 samples from 35 farms across nine German states were analyzed for MVV antibodies with an enzyme-linked immunosorbent assay (ELISA). Individual seroprevalence and clinical prevalence were 3.5% (30/849) and 13.3% (4/30), respectively. Seropositive sheep were detected in six flocks, with prevalences between 5.3 and 37.5%. Flocks in Eastern Germany and sheep older than 3 years showed the highest seroprevalence. Purchase of ewes was the main environmental risk factor for seropositivity. ELISA-based serotyping of SRLV in the MVV-positive sheep identified, besides genotype A, for the first time in Germany genotype B, whereas molecular genotyping, based on Sanger sequencing, identified only genotype A (A1, A3, A21, and A21-like). Apart from the obvious inconsistencies between the results of these two methods, presence of different viral sero- or genotypes suggest multiple virus introduction routes. Sequencing of exons 2 and 3 of the transmembrane protein 154 gene (*TMEM154*) of 530 sheep revealed non-synonymous variation at codons 35, 44 (known to be in linkage with a frame-shift variant coded in exon 1), and 70. Analysis of association between the putatively protective *TMEM154* codon 35 genotype KK and MVV seropositivity showed only a tendency toward significance (*p* = 0.088), whereas seroprevalence was significantly (*p* = 0.001) lower in sheep exclusively carrying the K allele at codon 35 and/or the M allele at codon 44. In contrast, MVV seropositivity was significantly higher in sheep with one or two I alleles at codon 70 (*p* = 0.045). Despite a low seroprevalence in the RPL breed in Germany, regular MVV monitoring is strongly recommended to avoid its spread. An eradication program based on serological testing and removal of seropositive sheep should be implemented. Selection for certain *TMEM154* alleles in affected flocks could support eradication measures, but their effect on MVV susceptibility in this breed requires further investigation.

## 1 Introduction

Maedi-visna (MV), originally described as a sheep-associated slow virus infection, together with caprine arthritis and encephalitis (CAE) in goats, forms the group of small ruminant lentiviruses (SRLV). The genus lentivirus belongs to the non-oncogenic retroviruses, which also include human (HIV) and feline (FIV) immunodeficiency viruses (1). In contrast to these immunodeficiency viruses, maedi-visna virus (MVV) does not infect T-cells and has a tropism for cells of the monocyte–macrophage lineage (2). Replication of the virus in macrophages may lead to inflammatory lesions in the lungs, joints, mammary glands, and brain (3, 4). Clinical signs such as dyspnea, dry cough, chronic weight loss, lameness, or mammary induration appear at the earliest 1–3 years after infection and sometimes do not manifest at all, indicating a subclinical infection (5–7).

MV is a persistent infection and no vaccines or treatment options are available yet (8). The main route of transmission is via the colostrum or milk of an infected ewe to its offspring (9). Horizontal transmission through close contact is another important route and has also been described between sheep and goats (10, 11). To prevent and eliminate the spread of MVV infection, regular monitoring of flocks, early detection, and culling of infected animals are essential (12, 91). In large flocks with high seroprevalences, motherless rearing of lambs is also a successful but costly sanitation option (13). Another cost-effective prevention and eradication strategy could be breeding for less MVV susceptible sheep (14). Some breeds, such as Texel and East Frisian milk sheep, are known to be highly susceptible to MVV, while in other breeds, such as Merinoland sheep, MVV-positive animals were rarely observed (15). These breed differences suggest that genetic factors play an important role in MVV susceptibility. It was reported that variants of the ovine transmembrane protein 154 gene (*TMEM154*) are associated with the serological MV status (16) and/or provirus concentration (17). Haplotypes 1–4 are the most common *TMEM154* haplotypes in sheep, and all four affect MVV susceptibility (16, 18). Sheep with one copy of either haplotype 2 or 3 (ancestral), both encoding glutamate (E) at position 35, were associated with an increased risk of MVV infection, while sheep homozygous for the derived haplotype 1 with lysine (K) at position 35 were less susceptible (16). Also in German sheep flocks with specific breed backgrounds (Texel, Brown Hair Sheep, East Frisian Milk Sheep, and Lacaune), animals with codon 35 genotypes EK and EE showed a significantly higher risk of being seropositive under MVV infection pressure than sheep with the genotype KK (19, 20). A protective effect was also observed for *TMEM154* haplotype 4, defined by a frameshift mutation (A4Δ53) in exon 1, representing a naturally occurring knock-out allele, so that alternate homozygotes do not have a functional TMEM154 protein (16, 21, 22). The A4Δ53 variant is in complete linkage with a threonine to methionine exchange (T44M) encoded in exon 2. Haplotype 2 differs from the ancestral haplotype 3 only by a single amino acid substitution from asparagine to isoleucine at position 70 (N70I). Although both haplotypes (2 and 3) were previously shown to be associated with increased susceptibility to MVV, Arcangeli et al. (23) were the first to report a significantly higher risk for sheep carrying one or two copies of haplotype 2 (codon 70 genotypes IN and II) compared to sheep without (NN).

However, not only host genetics but also pathogen genetics is an important risk factor for MVV infection (24, 25). SRLV are divided into four genotypes (A, B, C, and E), which differ from each other by 25–37% in their nucleotide sequences. The fifth putative group D has been reclassified as genotype A, but is still used by many authors (12). Genotype A is a large heterogeneous group with 27 up-to-date subgenotypes containing MVV-like strains. Genotype B, with five subgenotypes, includes CAEV-like strains (26). Both genotypes have been shown to be able to cross the species barrier and to be distributed worldwide in sheep and goats (11, 27). The other two genotypes are geographically restricted to small ruminants in Norway (genotype C) and goats in Italy (genotype E with 2 subgenotypes) (28–31). However, due to high variability of SRLVs, new variants are constantly emerging as more strains are analyzed (32).

Maedi (Icelandic word for dyspnoea) visna (Icelandic word for wasting) is named after the two typical clinical forms that were first described in Iceland in the 1930s after an import of Karakul rams from Germany (33). Progressive pneumonia, named maedi, is the most common form (34). In contrast, visna, a demyelinating leukoencephalomyelitis, is described only rarely (35, 36). Today, Iceland, Australia, and New Zealand are the only MVV-free countries. Except for these islands, the virus is distributed worldwide (33, 37) and is causing major economic losses in many countries (38). In 2005, Graber and Ganter (39) performed a nationwide survey of the epidemiological features of SRLV infections in Germany, including data from sheep and goat breeding associations and livestock health services. Results showed that only 0.21% of the German sheep population (0.93% of sheep flocks) and approximately 4.2% of the goat population had an accredited MV or CAE negative status. Another study by Hüttner et al. (40) observed a herd prevalence of 51.2% in their representative MVV screening study in Mecklenburg-Western Pomerania. These results show that the MVV is also widespread in Germany, particularly in the Texel breed, but can also occur in other breeds such as Merinoland and East Friesian milk sheep (15).

Pomeranian Coarsewool Sheep (in German ´Rauhwolliges Pommersches Landschaf´, RPL) is an old German land sheep breed that originated from Pomerania and was first named RPL in 1860 (41). In 1982, the breed was almost extinct and a conservation-breeding program was started with 46 ewes, 4 yearlings, and 7 rams (42, 43). Later, the S line, which came from a breeder in southern Germany, was added to the seven existing ram lines (44). The number of RPL pedigree sheep is steadily increasing and the Central Documentation of Animal Genetic Resources in Germany registered almost 3,200 RPL sheep in 2022 (45). RPL is a robust and resilient breed that is less susceptible to certain diseases (46, 47), but there are no data available on its susceptibility to MVV. The first MVV-positive RPL flock was detected by Hüttner et al. (40) in 2010. Sporadic serological investigations in other RPL flocks revealed negative results (unpublished data). The aim of this study was to determine the current seroprevalence of MVV in the RPL sheep population in Germany using an enzyme-linked immunosorbent assay (ELISA) and to identify both environmental and genetic risk factors for seropositivity, focusing on SRLV genotypes (pathogen genetics) and *TMEM154* genotypes (host genetics).

## 2 Materials and methods

### 2.1 Ethics approval statement

Before implementation, all animal procedures, beyond those only included in the voluntary MV monitoring of sheep flocks, were reviewed and approved by the respective state veterinary offices of the different German states involved (approval numbers: Baden-Württemberg 35-9185.82/A-11/23 (8 August 2023), Brandenburg LAVG-V6-2347-19-2023- 23-E (7 August 2023), Hesse V 54 19 c 20 15 h 01 Nr. V 3/2023 (21 August 2023), Lower Saxony 33.19-42502-04-23-00387 (4 August 2023), Mecklenburg-Western Pomerania 7221.3-2-012/23 (5 September 2023), North Rhine-Westphalia 81-02.04.40.2023. VG036 (11 August 2023), Saxony 25-5131/564/17 (9 October 2023), Saxony-Anhalt 203.m-42502-6- 011_TiHo_G (25 September 2023), Schleswig Holstein IX 552-117467/2023 (56-9/23 V) (25 September 2023)). The handling and sampling of the sheep followed European Union guidelines for animal care and handling, as well as Guidelines for Good Veterinary Practices.

### 2.2 Sample collection and DNA extraction

**Figure 1:**
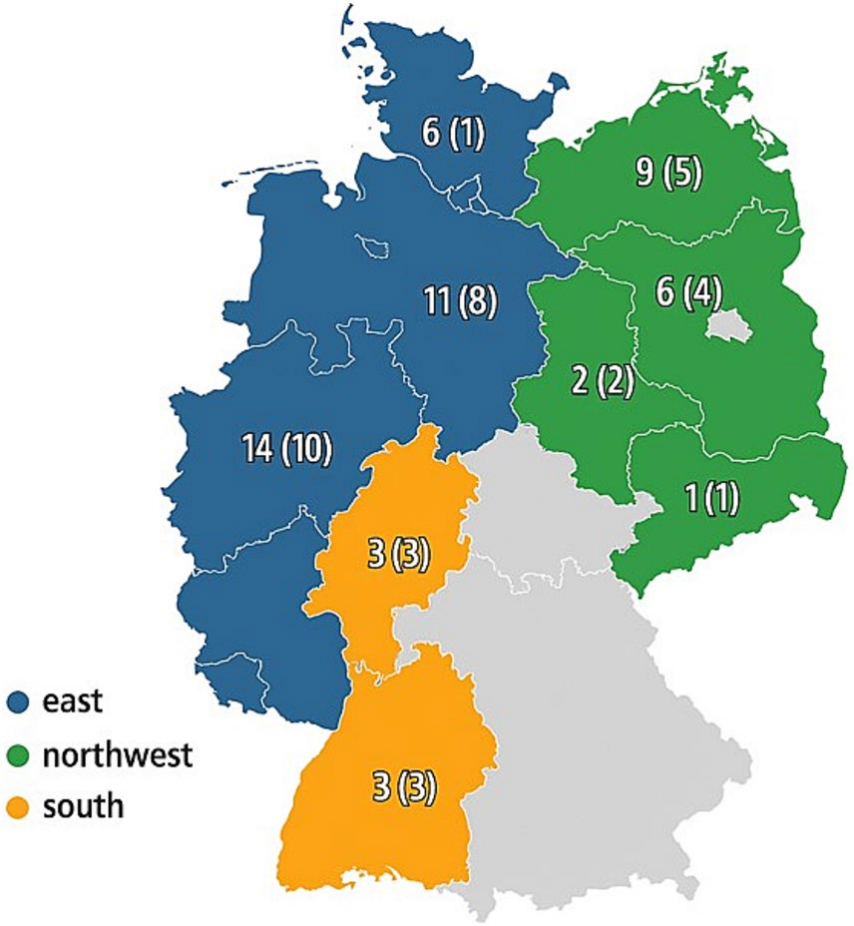
Location and numbers of the RPL flocks sampled in nine different states in Germany. Numbers without brackets: TMEM154 genotyped flocks; Numbers in brackets: TMEM154 genotyped and MVV monitored flocks; Blue: Northwestern Germany; Green: Eastern Germany; Orange; Southern Germany.

Between August and December 2023, blood samples were taken by veterinarians using 9 mL EDTA monovettes from jugular vein or vena cava cranialis of RPL sheep. The latter method is especially used in unshorn sheep (48). The study involved 54 flocks from nine German states, which were divided into three different study areas: North-western Germany (Schleswig Holstein, North Rhine-Westphalia, Lower Saxony), Eastern Germany (Saxony, Saxony-Anhalt, Brandenburg, Mecklenburg-Western Pomerania), and Southern Germany (Hesse, Baden-Württemberg) (number of sampled flocks per German state is given in Figure 1). In each flock, all breeding rams over 1 year of age and 1–10 unrelated females per ram line (no first- or second-degree relatedness) were sampled based on pedigree information, resulting in a total of 530 samples for sequence analysis of *TMEM154* exon 2 and 3.

Additionally, 35 of these flocks participated in the voluntary, but reportable, MV monitoring (Table 1). For this purpose, all RPL sheep over 1 year of age (plus four sheep aged 10 months) were sampled per flock, resulting in a total of 849 samples for MVV serology. The average flock size of the MVV-tested flocks was 52 RPL sheep (range: 7–295) (Table 1).

Combining the data from both analyses, a total of 395 RPL were tested for both *TMEM154* genotyping and MVV serology.

**Table 1:** Number and percentage of MVV-tested flocks and sheep in the three study areas.

Study area	Number of flocks	Number of RPL sheep	Number (%) of tested samples	Number (%) of positive sheep
Northwestern Germany	19	647	324 (50.1)	2 (0.9)
Eastern Germany	12	1,076	473 (44.0)	28 (5.7)
Southern Germany	4	91	52 (57.1)	0 (0.0)
Total	35	1841	849 (46.1)	30 (3.5)

EDTA-blood samples were stored at <+8 °C after collection for up to 5 days. Blood samples for MVV serology were centrifuged and plasmas were separated before freezing at −20 °C until use. DNA was extracted from whole blood using the NucleoMag Blood 200 μL kit (Machery-Nagel GmbH & Co. KG, Dueren, Germany), which is based on magnetic separation by reversible adsorption of nucleic acids to paramagnetic beads under appropriate buffer conditions. DNA quality and quantity were measured with a Nanodrop ND-1000 spectrophotometer (Thermo Fisher Scientific Inc., Waltham, MA, USA). DNA samples were stored at −20 °C until use.

### 2.3 Questionnaire

A questionnaire was developed and sent to each RPL breeder of the 54 flocks to be completed before, during, or after the farm visit for blood sampling. The aim of the questionnaire was to obtain flock-level information including flock size, pedigree breeding, management practices (type of housing, mating, lambing, and lamb rearing), sheep purchases, presence of other sheep breeds or goats on the farm, health measures (such as vaccinations and prevention of ecto- and endoparasites), and potentially known MV status. However, only the 35 questionnaires corresponding to flocks tested serologically for MVV were used for statistical risk analysis.

### 2.4 MVV serology

All plasma samples were tested by using a commercial competitive ELISA test kit (VMRD Inc., Pullman, Washington, USA), according to the manufacturer’s instructions. Based on the optical density (OD) results, the percent inhibition (PI) was calculated as follows:


$$\mathrm{PI}=100[1-(\frac{\text{Sample}\;\mathrm{OD}}{\text{Negative Control}\;\mathrm{OD}})]$$

According to the manufacturer’s recommendations, a sample was classified as seropositive when it produced ≥ 35% inhibition.

Although based on the CAEV envelope glycoprotein, the sensitivity and specificity of the assay were 98.6 and 96.9%, respectively, when used with sheep sera (49).

### 2.5 SRLV serotyping and genotyping

Twenty-nine of the 30 seropositive plasmas were serotyped with the indirect Eradikit ELISA for SRLV genotyping (Eradikit SRLV Genotyping IN3 Diagnostic, Torino, Italy). The genotyping ELISA plates are coated with specific recombinant subunits of genotypes A, B, and E on three separate strips. Genotype-specific antibodies bind to the corresponding antigen and lead to colour development. To determine a positive result for one genotype, the measured OD value must be > 0.4, and 40% above the other OD values. The results were classified as indeterminate if the OD values of all genotypes were < 0.4, or if the difference between the two most reactive antigens was too low (< 40%). In the latter case, a double infection could be possible, according to the manufacturer’s instructions.

To obtain more precise and comprehensive data, a nested end-point PCR amplification of the SRLV *gag-pol* region was carried out with DNA samples from all seropositive sheep. In the first round, a 1,300 bp fragment was amplified. In the second round, an 800 bp fragment was obtained using the first-round amplified product as template. The following set of primers was used: GAGF1 for 5′-TGGTGARKCTAGMTAGAGACATGG-3′ and POLR1 rev 5′-CATAGGRGGHGCGGACGGCASCA-3′ in the first round, and GAGF2 for 5′-CAAACWGTRGCAATGCAGCATGG-3′ and POLR2 rev 5′-GCGGACGGCASCACACG-3′ in the second round. The first-round of the nested PCR was carried out in 50 μL reaction volume consisting of 5 μL of Buffer 10X, 2 μL of MgCl2 25 mM, 1 μL of dNTPs 10 mM (ThermoFisher Scientific, Massachusetts, USA), 1.5 μL of each primer (20 μM), 5 μL of BSA 10X, 0.2 μL of Taq Platinum DNA Polymerase (Invitrogen Corporation, USA) and 3 μL of cDNA or 10 μL of DNA. The PCR program used had the following thermal profile: Taq polymerase activation at 94 °C for 2 min, followed by 40 cycles with denaturation at 94 °C for 45 s, annealing at 55 °C for 1 min, and extension at 72 °C for 1 min, followed by 10 min at 72 °C. The second round of the nested PCR was carried out in 50 μL reaction volume consisting of the same volumes from the first-round PCR and 3 μL of template. The PCR program used had the following thermal profile: Taq polymerase activation at 94 °C for 2 min, followed by 40 cycles with denaturation at 94 °C for 45 s, annealing at 65 °C for 1 min, and extension at 72 °C for 1 min, followed by 10 min at 72 °C. Both first and second-round mix volumes were adjusted using DEPC treated water. Results were analyzed via electrophoresis on a 2% agarose gel, using Midori Green Advance (NIPPON Genetics Europe GmbH, Düren, Germany) as an intercalating agent. Only samples that yielded sufficient quantities of PCR products were subjected to Sanger sequencing. Bands of the expected size of 800 bp were excised from agarose gel and purified using the QIAquick Gel Extraction kit (Qiagen, Hilden, Germany) following the manufacturer’s protocol. The sequencing reaction was conducted using the BrilliantDye Terminator Cycle Sequencing v3.1 Kit (NimaGen BV, Nijmegen, The Netherlands). The sequencing process was replicated twice for each sample using the forward primer GAG-F2 and the reverse primer POL-R2, both used at a 3.2 μM concentration. The following thermal profile was used for the sequencing reaction: Taq polymerase activation at 96 °C for 45 s, followed by 25 cycles with denaturation at 96 °C for 10 s, annealing at 50 °C for 5 s and extension at 60 °C for 4 min. Precipitation of sequencing reaction products was done with the DyeEx 2.0 Spin Kit (Qiagen, Hilden, Germany) following the manufacturer’s protocol and denatured at 95 °C for 5 min after the addition of HiDi Formammide (Applied Biosystems Life Technologies, Warrington, UK). Following denaturation, the products were loaded into the ABI PRISM 3130 Genetic Analyzer automatic sequencer (Applied Biosystems, Foster City, CA, USA). Consensus sequences for each sample were obtained using DNAStar Navigator software v.15, then compared and aligned with other SRLV sequences downloaded from GenBank, including references for subgenotypes. The alignment was done with Clustal X v.2 and was further refined and trimmed using BioEdit v.7.2.5 (50). The phylogenetic tree was obtained with MEGA X v.10.2.6, using Maximum Likelihood method and 1,000 bootstrap replicates, and further refinement has been done with ITOL online tool (https://itol.embl.de/).

### 2.6 Determination of *TMEM154* genotypes

PCR amplification and Sanger sequencing of *TMEM154* exon 2 and 3 were performed using extracted DNA from 530 sheep samples as described by Heaton et al. (16). Sequencing reactions and electrophoresis were carried out by LGC Genomics GmbH (Berlin, Germany). Sequence chromatograms were analyzed using the software ChromasPro (Technelysium Pty Ltd., South Brisbane, Australia) for DNA and protein sequence variants.

### 2.7 Statistical analysis

The data were stored and organized in Microsoft Excel 2016 (Microsoft Corporation, Redmond, USA). Associations between potential risk factors and individual and flock-level seropositivity were assessed by using a chi-square or Fisher’s exact test and by calculating the odds ratio (OR) to facilitate comparison with results from other breeds. Due to the small number of positive samples, only a univariable analysis was performed for each independent variable. SAS Studio Version 9.4 program (SAS Institute Inc., Cary, NC, USA) was used for the statistical analyses.

## 3 Results

### 3.1 MVV seroprevalence in RPL flocks

Among a total of 849 RPL sheep plasma samples tested with the competitive ELISA, 30 showed a positive result (3.5%). Seropositive animals were detected in six flocks (17.1%), one of them was located in the northwest, and the others in the east of Germany (Table 1; Figure 1). The within-flock prevalence in positive flocks ranged from 5.3 to 37.5%. Four out of 30 seropositive sheep developed typical clinical signs, thus clinical prevalence was 13.3% (Table 2).

**Table 2:** Seroprevalence and clinical prevalence in MVV-positive RPL flocks.

MVV-positive flocks	Positives (*n*)	Seroprevalence (%)	Type and number (*n*) of clinical symptoms	Clinical prevalence (%)
Flock 1	2	15.4	Nasal discharge and dry cough (1)	50.0
Flock 2	8	5.3	–	0.0
Flock 3	7	11.7	Cachexia (1)	14.3
Flock 4	7	12.3	–	0.0
Flock 5	3	37.5	Cachexia and Arthritis (1) Arthritis (1)	66.7
Flock 6	3	37.5	–	0.0
Total	30	19.9	4	21.8

–: no clinical symptoms.

### 3.2 Risk factors of individual MVV seropositivity

In the group of sheep older than 3 years, significantly more sheep were seropositive (4.8%) than in the group of sheep younger than 3 years (1.5%) (Table 3). Seroprevalence among sheep from the eight different ram lines was not significantly different (*p* = 0.37). Most seropositive sheep were found in ram line 3 (7.14%). A portion of 13.3% of the tested RPL sheep could not be assigned to any specific ram line, including seven seropositive sheep (6.2%).

**Table 3:** Results of the statistical analysis for individual risk factors associated with MVV seropositivity.

Risk factor	Category	*n*	Seropositive (%)	OR	CI 95%	*p*-value
Age*	≤ 3 years	334	1.5	reference		
	≥ 3 years	480	4.8	3.312	1.246–8.801	0.011
Sex	female	740	3.2	reference		
	male	109	5.5	1.738	0.694–4.353	0.26

*: 2 out of 35 sheep with missing data were seropositive (5.7%).

OR: odds ratio.

CI: confidence interval.

### 3.3 Risk factors of MVV seropositivity at flock-level

All the participating farmers kept sheep for breeding (100%), with 74.3% also using them for landscape conservation, and 42.9% keeping them as a hobby. The only two management systems of the participating RPL farms were paddock farming and sheep transhumance.

**Figure 2:**
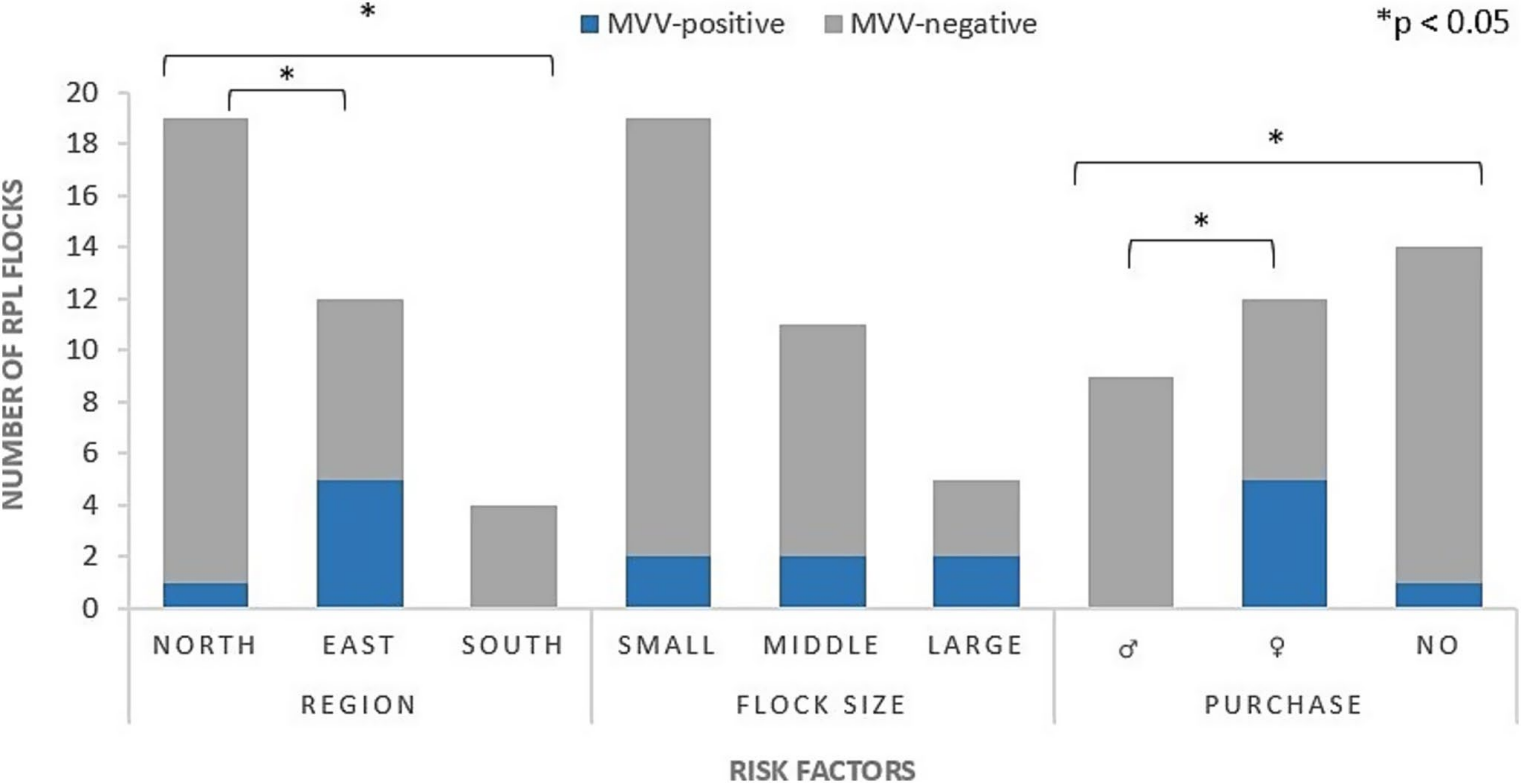
Numbers of MVV-positive or negative RPL flocks in relation to three different risk factors. Region: north (northwest), east, south (Figure 1); Flock size: small (1–30 sheep), middle (31–100 sheep), large (>100 sheep); Purchase: ♂ (only ram(s)); ♀ (ewe(s) and/or female yearling(s)); no (no purchase).

In large positive flocks (>100 sheep), seroprevalence was 40% and therefore higher than in medium-sized (31–100 sheep) (18.9%) and small (1–30 sheep) (10.5%) flocks (Figure 2). Contact with other sheep breeds or the presence of goats in RPL flocks also tended to increase the prevalence (Table 4). However, none of these differences were statistically significant.

In eastern Germany, five out of twelve flocks (41.7%) had a serologically positive flock status (Figure 2). Therefore, region was a significant risk factor at flock-level (*p* = 0.03). The addition of new RPL sheep in the last year was also significantly associated with a MVV-positive flock status (*p* = 0.02) (Figure 2). Especially the purchase of female sheep increased the odds of infection by 15.7 times (Table 4).

**Table 4:** Results of the statistical analysis for risk factors associated with MVV seropositivity at flock-level.

Risk factor	Category	*n*	Seropositive (%)	OR	CI 95%	*p*-value
Contact with other sheep breed(s)	No	23	8.7	reference		
	Yes	12	33.3	5.25	0.799–34.496	0.151
Presence of goats	No	26	15.4	reference		
	Yes	9	22.2	1.571	0.235–10.491	0.635
Purchase (all)	No	14	7.1	reference		
	Yes	21	23.8	4.063	0.42–39.257	0.366
Purchase of only ewe(s)/yearling(s)	No	23	4.4	reference		
	Yes	12	41.7	15.714	1.561–158.211	0.012

OR: odds ratio.

CI: confidence interval.

### 3.4 SRLV serotypes and genotypes

Of the 29 plasma samples serotyped using the Eradikit ELISA, 37.9% were positive for one of the defined genotypes A (three samples) or B (eight samples), while 55.2% could not be assigned to any genotype (Table 5). Two sera (6.9%) showed a possible co-infection.

**Table 5:** SRLV serotyping (*n* = 29) and genotyping (*n* = 13) results of 30 seropositive RPL sheep and their *TMEM154* E35K, T44M, and N70I genotypes.

Flock	Sheep (*n*)	Eradikit ELISA	Sanger sequencing	*TMEM154* Genotype		
				E35K	T44M	N70I
1	1	B	A21-like	EK	TT	IN
	2	B	A21-like	EK	TT	NN
2	1	A/E	A3	EE	MT	NN
	2	Indet	–	EK	TT	NN
	3	A	A3	EK	TT	IN
	4	B	–	EE	MT	NN
	5	A	–	EK	TT	NN
	6*	Indet	–	EK	MT	NN
	7*	Indet	–	KK	TT	NN
	8	Indet	–	EK	TT	NN
3	1	A	–	EK	TT	IN
	2	Indet	–	EE	TT	II
	3	B	A21	EK	TT	IN
	4	B	A21	EK	TT	IN
	5	Indet	–	EK	TT	IN
	6	B	A21	EK	TT	IN
	7	Indet	–	EK	TT	IN
4	1	Indet	–	EE	MT	NN
	2*	Indet	–	KK	TT	NN
	3	Indet	A21	EK	TT	NN
	4	Indet	A21	EK	TT	NN
	5*	Indet	A21	KK	TT	NN
	6	Indet	A21	EE	MT	NN
	7	Indet	–	EK	TT	NN
5	1	Indet	–	EE	TT	IN
	2	Indet	–	EE	TT	IN
	3	–	A1	EK	TT	NN
6	1*	B	–	EK	MT	NN
	2*	B	–	KK	TT	NN
	3	A/B	A˟	EK	TT	IN
Total		29	13			

Indet: indeterminate results.

–: not tested; *: putatively MV-resistant genotype combinations; ˟: subgenotype unclassified.

**Figure 3:**
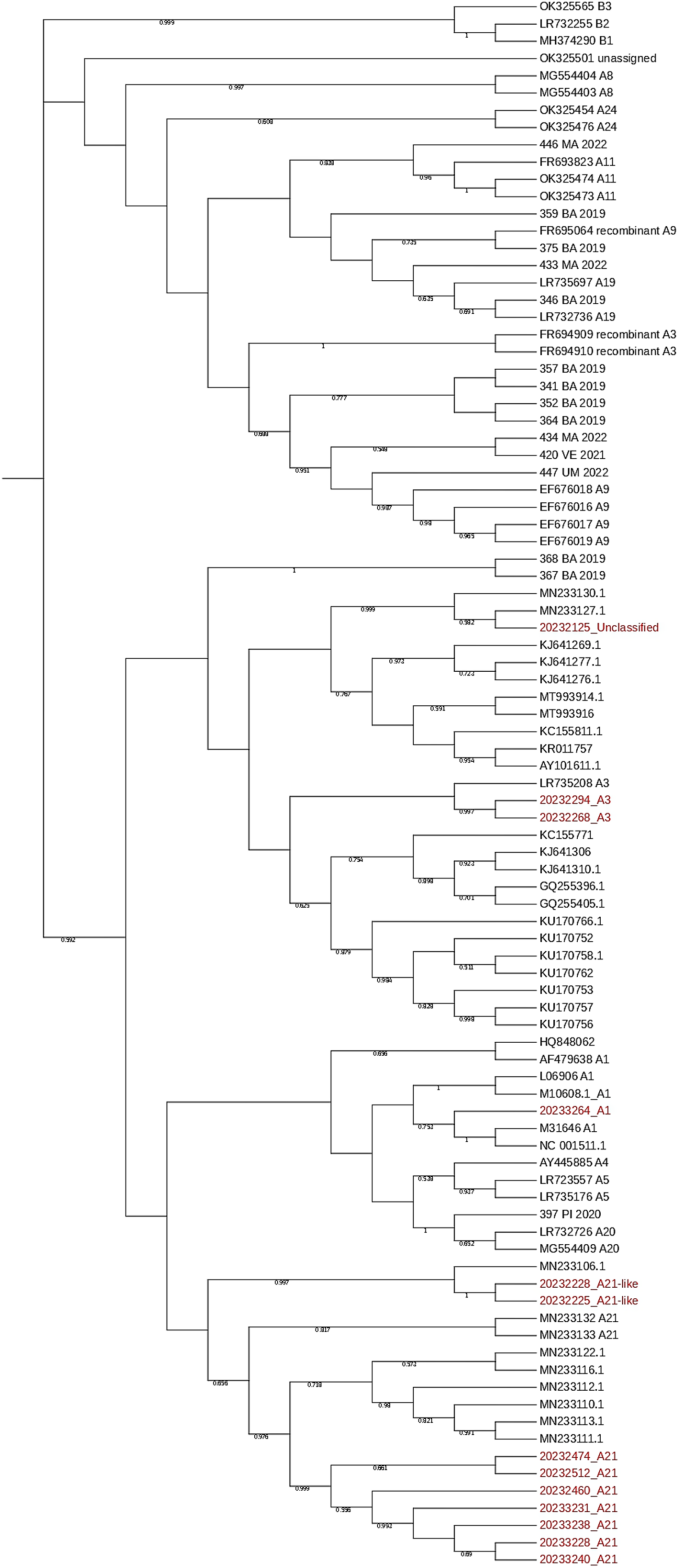
Phylogenetic tree including SRLV sequences generated in this study (red) and sequences downloaded from GenBank (black), including references for subgenotypes.

Thirteen out of 30 seropositive sheep samples were Sanger sequenced. In contrast to the Eradikit ELISA results, virus genotyping by DNA sequencing revealed only the SRLV genotype A, comprising three different subgenotypes, in these sheep: A1 (*n* = 1), A3 (*n* = 2), and A21 or A21-like (n = 9). For one sample, the subgenotype was not classified, because its phylogenetic group was not clear. However, also this sample belonged to the SRVL genotype A group (Table 5; Figure 3). Twelve samples were simultaneously serotyped and genotyped. Only one animal showed concordant results (8.33%). In both cases where the Eradikit ELISA indicated a potential co-infection, SRLV genotyping confirmed the presence of genotype A only. Out of the remaining nine discrepant samples, five were serotyped as SRLV serotype B, and four produced indeterminate serotyping results (Table 5).

### 3.5 *TMEM154* genotypes and results of association analyses

**Figure 4:**
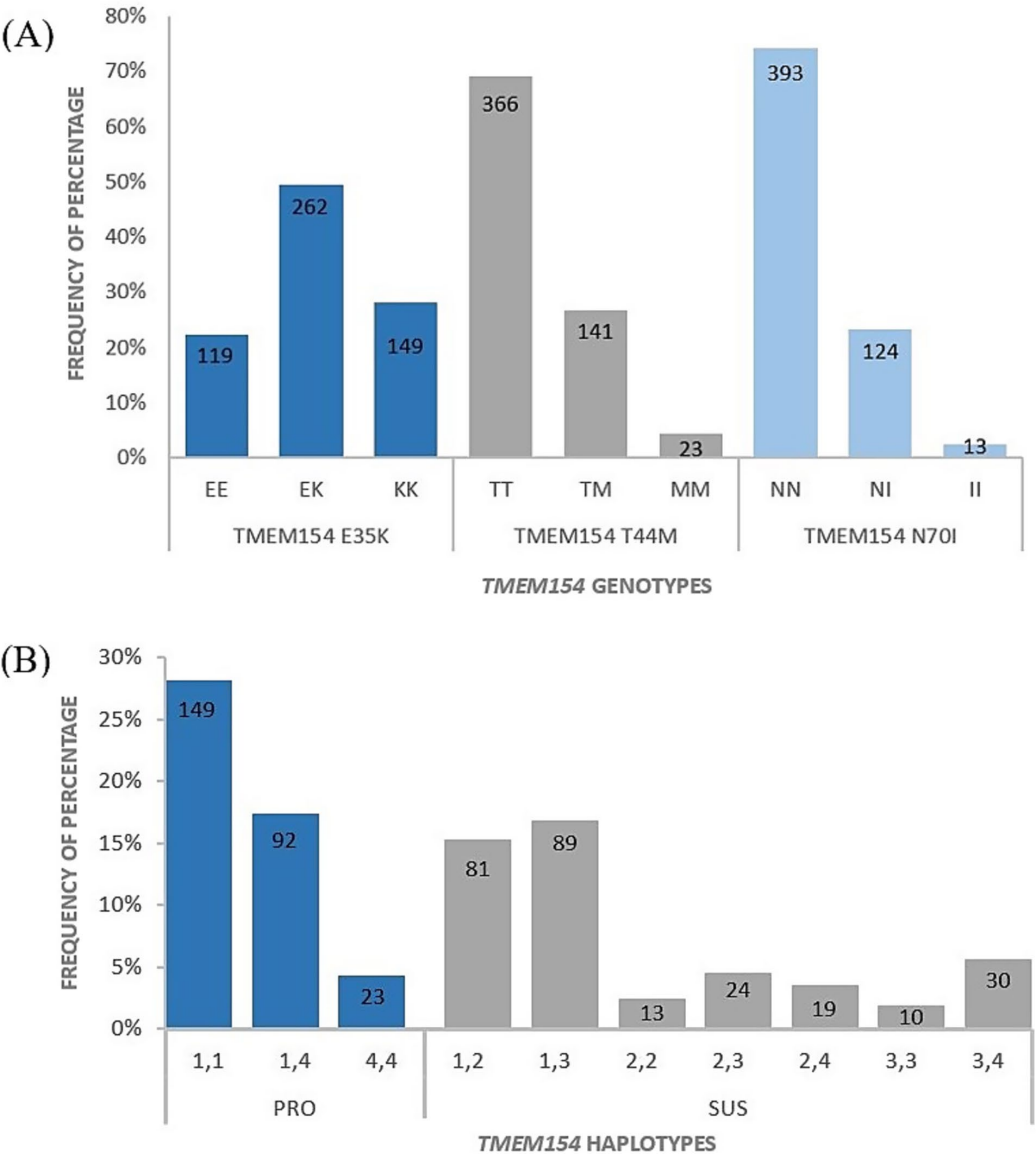
Percentage distribution (*y*-axis) and absolute animal counts (within bars) of *TMEM154* E35K, T44M, and N70I genotypes **(A)** and *TMEM154* haplotypes **(B)** among 530 genotyped RPL sheep.

Sequencing of *TMEM154* exons 2 and 3 identified variations at codons 35, 44, and 70. All other positions were monomorphic in the investigated 530 sheep. At codon 35 genotype EK occurred at highest frequency (49%), followed by KK (28%) and EE (23%) (Figure 4A). The frequencies of the putative protective allele K and risk allele E were 53 and 47%, respectively. Allele M at codon 44 was present at a frequency of only 18%, whereas allele T was found in 82% of the animals. At codon 70, allele N was observed in 86% of the sheep, while allele I occurred in 14%. The distribution of the different *TMEM154* genotypes and the resulting haplotypes among the 530 genotyped RPL sheep is shown in Figure 4.

In MVV-tested flocks, 28% of serologically negative sheep (*n* = 101) carried the putatively protective genotype KK, whereas this genotype was present in 13% of the positive sheep (*n* = 4) (Table 6). This difference tended to be significant (*p* = 0.088).

**Table 6:** TMEM154 E35K, T44M, and N70I genotype frequencies of the 395 MVV tested RPL sheep with the statistical results.

TMEM154	Genotype	MV result (%)		OR	CI95%	*p*-value
		Positive	Negative			
E35K	EE, EK	26 (86.7)	264 (72.3)	2.487	0.848–7.303	0.088
	KK	4 (13.3)	101 (27.7)			
T44M	TT, TM	30 (100)	345 (94.5)	–	–	0.385
	MM	0 (0.00)	20 (5.5)			
N70I	NI, II	12 (40.0)	86 (23.6)	2.163	1.002–4.668	0.045
	NN	18 (60.0)	279 (76.4)			
E35K- T44M	SUS	24 (80.0)	178 (48.7)	4.202	1.678–10.521	0.001
	PRO	6 (20.0)	187 (51.2)			

PRO: putatively MV-resistant genotype combinations (35 KK/44 TT, 35 EE/44 MM, 35 EK/44 MT).

SUS: putatively MV-susceptible genotype combinations (35 EE/44 TT, 35 EE/44 MT, 35 EK/44 TT).

OR: odds ratio.

CI: confidence interval.

–: no data available.

Although all 20 individuals homozygous for allele M at codon 44 (MM), and thus homozygous for the linked, putatively protective A4Δ53 frameshift mutation, were tested serologically negative for MVV, this association was not statistically significant (Table 6). However, the exclusive presence of codon 35 allele K and/or codon 44 allele M showed a statistically significant protective effect (*p* = 0.001). Conversely, codon 70 allele I was significantly associated with susceptibility (*p* = 0.045).

All other association analyses of *TMEM154* genotypes, whether compared individually or grouped (EE vs. EK/KK, TT vs. TM/MM, and II vs. NI/NN), revealed no statistically significant results and were therefore not discussed further in this study.

## 4 Discussion

This study, the first conducted in Germany, sheds light on seroprevalence of MVV infection in the old, native sheep breed RPL across nine out of sixteen German states, while also investigating potential risk factors and genetically characterizing the circulating SRLV. Therefore, it combines epidemiological, genetic, and molecular insights to provide valuable data for future control strategies. The 3.5% individual and 17.1% flock seroprevalences were considerably lower than the flock prevalence of 51.2% reported in the representative study by Hüttner et al. (40) in sheep flocks in the federal state of Mecklenburg-Western Pomerania. Among other European countries, only Belgium had a similar herd prevalence with 17.2%, but individual seropositivity (9.4%) was higher (51), such as individual seroprevalence in Austria (9.7%), Croatia (10%), Poland (17.3%), and Spain (24.8%) (52–55). The only countries with a lower reported individual seroprevalence were Brazil with 1.07% and Costa Rica with 1.95% (56, 57). According to Straub et al. (15), coarse-wool sheep may be more susceptible to develop clinical MV symptoms than fine-wool sheep. So far, this hypothesis could not be confirmed by the present study, as only four out of 30 positive RPL sheep (13.3%) showed MV typical clinical signs, such as dry cough, cachexia, lameness, or swollen carpal joints (7). Villagra-Blanco et al. (57) detected a higher clinical incidence of 57%, although the individual seroprevalence was lower. However, the actual clinical prevalence could be higher, as it was only a snapshot of the veterinarians at the farm visit or an assessment by the breeders. Additionally, the majority of infected animals were relatively young. In general, the disease occurs in sheep older than 3 years, but in this study, a seropositive yearling showed a dry cough and nasal discharge, which might also be a result of secondary bacterial infection (7).

Nevertheless, when interpreting the obtained seroprevalence in this breed, certain limitations should be taken into account. Besides the possible impact of seroconversion, seroreversion, particularly in sheep older than 3 years, may also lead to false-negative results and consequently to an underestimation of MVV prevalence. Kalogianni et al. (58) observed seroreversion in 8.6% of previously MVV-positive sheep across five farms over 2 years, suggesting a possible suppression of neutralizing antibodies associated with poor general health or individual variations in the humoral immune response, although the exact mechanisms remain unclear. Furthermore, the imperfect sensitivity of the ELISA test used may have contributed to additional misclassification (49). Taken together, due to such factors the actual infection rate in the RPL breed may be higher than the serological data suggest, which should be considered when interpreting results and developing effective control or breeding programs.

### 4.1 Environmental risk factors for MVV seropositivity at individual and flock-level

In this study, age was the only significant individual risk factor for seropositivity (*p* = 0.01). Sheep older than 3 years had a threefold higher risk of being serologically MV-positive than younger sheep. The fact that MVV infection rises with animal age, with a peak between 3 and 5 years, has already been established in many other studies (55, 59–61). This is not surprising, as the opportunity for virus exposure, infection with the virus, and antibody production against the virus increases with advancing age.

The purchase of new positive animals in the last year was observed as main risk factor at flock-level for the introduction of MVV into negative RPL flocks (*p* = 0.02). This result supports similar findings in other studies (55, 62). Interestingly, five of the six positive flocks also purchased female sheep, whereas the integration of foreign rams alone was not associated with MVV-positive flock status. A possible reason for this could be the more intensive MV monitoring of RPL rams, which is required for participation in certain exhibitions. Another explanation might be a predominantly lactogenic transmission of MVV (9), as some of the purchased ewes or their progeny were seropositive. The fact that some of the seropositive sheep were the offspring of MVV-seronegative ewes speaks against an exclusively lactogenic transmission. This assumption is supported by recent studies (10, 63). The finding that the seroprevalence was highest in large (40%) compared to medium (18%) and small-sized flocks (11%) also argues in favor of horizontal transmission, as direct close contact and limited space are known risk factors (64). However, it is important to note the low number of large RPL flocks in this study. Previous studies by Lago et al. (55) and Hüttner et al. (40) also reported that the likelihood of MVV seroprevalence increased with flock size. Given that the largest RPL flocks are located in their original home in an eastern federal state of Germany, it is not surprising that the highest number of positive RPL sheep (5.7%) was identified in this region. Consequently, the three study areas showed a significant difference in the MVV seroprevalence (*p* = 0.03).

Direct contact with other sheep breeds was identified as a potential risk factor. Although seroprevalence in mixed-breed flocks was fivefold higher than in pure RPL flocks, this association was not significant (*p* = 0.15). Nevertheless, occasional introduction of MVV into the RPL breed through contact with MVV-positive sheep of other breeds cannot be entirely excluded. Unfortunately, data on the MV status of other sheep breeds were only available from one seropositive flock, in which all non-RPL sheep were tested seronegative. The presence of infected goats can also be considered a potential risk factor for the introduction of lentiviruses into the RPL breed, as several reports have shown that SRLV crossed the species barrier through direct contact (11, 55, 65). However, in our data, keeping of goats in RPL flocks was not significantly associated with their SRLV status (*p* = 0.64).

Our finding that rams were almost twice as often seropositive as ewes was not statistically significant (*p* = 0.26) and was likely due to random chance given the small number of seropositive rams. The fact that only the purchase of female sheep is a significant risk factor, along with previous studies (53, 60, 66) reporting higher prevalences in females than in males, supports this hypothesis. However, a possible reason for higher seroprevalence in males could have been the more frequent exchange of rams between flocks for breeding purposes and that they are often kept together with rams of other breeds, or goats.

### 4.2 SRLV genetics as risk factor for MVV seropositivity

Genetic variability of SRLV is an important tool for epidemiological studies and can provide information on the origin of the virus (67, 68). Moreover, genetic susceptibility of sheep to SLRV infection has been shown at least in some studies also to depend on the genotype of the present SRLVs (25, 69). Serological diagnostics, such as ELISA, serve as a first-line epidemiological tool to characterize SRLV serotypes (70, 71). However, a high variability of circulating strains represents a limitation of this method, as no current serological tests are capable of discriminating among all viral variants with certainty (72, 73). This limitation was confirmed in the present study, where the Eradikit ELISA yielded a high percentage (55.2%) of indeterminate results. In contrast, the more expensive and time-consuming genetic analysis by PCR and Sanger sequencing revealed no indeterminate results, and determined SRLV genotype A21 in four samples with indeterminate ELISA results. In two cases, sheep were serologically tested as coinfected with genotypes A/B and A/E, but only genotype A was detected by sequencing. This discrepancy may be due to the fact that SRLV strains show the ability to infect different organs or tissues in the same host. This phenomenon, called compartmentalization, has been described before (74–77). One probable explanation for the inability to confirm the co-infection is that only one type of tissue was used for Sanger sequencing. Additionally, a recent study showed that Sanger sequencing may have limitations compared to next-generation sequencing (NGS) when it comes to detecting co-infection when there is a low proviral load (78). In such cases, co-infections can be detected by cloning of PCR products before sequencing, but this was not possible in this study. It has also to be admitted that a limitation of SRLV genotyping by sequencing is the usually lower number of samples which can be genotyped compared to serological tests, as the needed minimal amount of provirus DNA usually cannot be obtained from all serologically positive samples (79, 80). However, co-infections with different SRLV genotypes have been reported previously (81, 82), whereas genotype E has only been detected in Italian goats so far (83). Colitti et al. (71) also identified genotype E in sheep using the Eradikit genotyping ELISA, whereas provirus sequencing determined genotype A. Therefore, in our study, we trust the sequencing result (A3) more than the ELISA result (A/E coinfection). By the way, A3 was also found in a second sheep of the same flock.

Due to the low concordance (8.33%) of the serotyping ELISA and genotyping PCR, these results should be interpreted with caution. Similar findings have been reported by Jiménez et al. (72), Colitti et al. (71), and Olech and Kuźmak (73) with concordance rates of only 25.7%, less than 40, and 42.3%, respectively. In contrast, a concordance of more than 97% between serotyping and genotyping was reported in the study by Nogarol et al. (70). Even though the number of samples compared in the present study was low (*n* = 12), the results suggest that serological serotyping with the Eradikit genotyping ELISA is not a reliable method for predicting the SRLV genotype in infected flocks, as described recently (73). This is of practical importance for eradication programs that focus on specific genotypes, such as in Switzerland, where only genotype B-infected goats are culled, given the considerable risk of inaccurate results (84). Up to now, this is not planned in any control program for MVV worldwide.

As genotype B was not detected by provirus sequencing and the only previous study on SRLV genotypes in German sheep populations (67) only identified A genotype, it appears unlikely that SRLV B genotypes are present in the analyzed RPL flocks. However, we recently identified for the first time SRLV genotype B in a German sheep flock by sequencing, but in another breed and geographical region (unpublished results). To date, no studies have specifically investigated RPL sheep in Germany, therefore we cannot exclude that our serological detection of genotype B may indicate a previously unrecognized presence of this genotype in the breed. Further testing using Sanger sequencing in RPL sheep from the same federal state and/or other regions would be necessary to prove this.

Regardless of whether the results of serotyping ELISA or of provirus sequencing are considered more reliable, both methods revealed viral heterogeneity in the RPL population. This suggests multiple origins and routes of introduction of the virus into the RPL breed and single flocks. In this regard, the provirus sequencing results even indicated a flock-specific SRLV distribution.

### 4.3 *TMEM154* genotype as risk factor for MVV seropositivity

Beyond the environmental factors, the low MVV infection prevalence in RPL sheep may indicate a low genetic susceptibility of this breed. Genotype KK at codon 35 of *TMEM154* gene was observed to confer lower susceptibility to MVV infection in various American, Asian, and European sheep breeds (18, 20, 85, 86). Also in the RPL breed, sheep with this genotype were less frequently seropositive compared to sheep with genotypes EK and EE, but this association was not statistically significant (*p* = 0.09). This could be explained by the low number of serologically positive sheep present in tested flocks (only 30 out of 395). Whether a higher number of serologically positive sheep would lead to a significant result needs to be tested in a further study, for which additional MVV-positive RPL flocks would have to be found. Increasing the sample size was not possible, as the mandatory reporting of MVV in Germany discouraged many voluntarily participating RPL breeders from testing their flocks for MVV. A missing significance of the KK genotype effect on MVV susceptibility, possibly due to low number of seropositive sheep, was also postulated in other studies involving other breeds (19, 69, 86).

The odds of infection for RPL sheep carrying one or two copies of *TMEM154* allele E at codon 35 were 2.5 times higher than those for sheep homozygous for the K allele (*p* = 0.09, OR = 2.487, CI 95% = 0.847–7.303). However, 13.3% (*n* = 4) of seropositive RPL sheep still carried the putatively protective genotype KK. Previous studies have already shown that the KK genotype does not provide full protection against MVV infection (16, 63). There are also hints that the protective effect of this variant could be breed-dependent. In a survey of German MVV-affected sheep flocks, a significant (even if not absolute) protective effect of the KK genotype was observed in Texel and some milk sheep breed flocks. By contrast, this was not observed in the Merinoland sheep breed, where the frequency of the KK genotype in seropositive sheep (75%) was even higher than in the seronegative animals (59%) (19). Further support for this breed-specific effect comes from a recent study by Materniak-Kornas et al. (22), in which none of the KK-genotyped Cameroonian sheep tested positive for MVV. This finding is consistent with the hypothesis that the protective effect of the KK genotype may be more pronounced in MVV-susceptible breeds such as Texel, East Friesian, and Cameroonian sheep, while being less effective in more resistant breeds like Merinoland and potentially also RPL sheep. Interestingly, the present study showed that 12 seronegative offspring originated from seropositive ewes. Ten of these offspring were born after 2020 and may still become seropositive due to the long incubation period. The remaining two offspring, both over 3 years of age (born before 2020), had *TMEM154* genotype KK, which in turn suggests a protective effect. Leymaster et al. (63) already reported this protective effect of genotype KK for the lactogenic but also for the horizontal transmission.

Haplotype 4, defined by a *TMEM154* frameshift mutation (A4Δ53) that represents a naturally occurring knock-out allele, has been in the past suggested to potentially offer complete MVV-resistance (16). However, due to its low frequency (less than 5%) in most sheep populations studied so far, association analyses with MVV susceptibility are rare (22, 23). In the RPL breed, the frequency of A4Δ53, determined by genotyping the linked variation at codon 44 in exon 2, is 17.6%. In 2015, Clawson et al. (87) showed for the first time that animals homozygous for this “knockout” allele could still be infected with MVV. Freking et al. (21) later confirmed this finding, although seropositive 4,4 sheep did not show clinical symptoms and had healthy-appearing lung tissues, suggesting that viral replication may be limited in these animals. In the present study, all 20 sheep homozygous for the A4Δ53 frameshift mutation were seronegative, and only six of the 106 heterozygous animals (5.7%) were tested seropositive. Although these results support the hypothesis of reduced susceptibility, statistical significance could not be established, likely due to the low number of infected animals, as discussed before for the variation E35K.

To the best of our knowledge, no study has analyzed until now the combined effect of the two *TMEM154* alleles, K at codon 35, and A4Δ53 (determined by M at codon 44), which were assumed to be (at least partially) protective. Indeed, in the RPL sheep, the frequency of seropositive sheep was significantly lower in carriers of exclusively these two alleles (genotypes 35KK, 44MM, or 35 EK in combination with 44 MT) and the odd of infection was 4.2 times higher for sheep without them (*p* = 0.001, OR = 4.202, CI 95% = 1.678–10.521). A total of 49% of the genotyped RPL sheep carried one of these three genotypes. This provides a promising basis for resistance-oriented breeding in this breed.

Haplotypes 2 and 3 differ only by a single amino acid substitution at position 70 of *TMEM154* (N70I) (16). In the past, both variants were considered functionally equivalent dominant risk alleles, with some studies reporting very high MVV prevalence rates among animals carrying either haplotype (16, 21), suggesting limited functional relevance of *TMEM154* N70I mutation. However, this assumption was later challenged by Arcangeli et al. (23), who showed that carriers of codon 70 genotypes NI or II had a significantly higher risk of infection than those with genotype NN. This is in accordance with the present study, in which a significantly higher susceptibility was observed for sheep with codon 70 genotypes NI and II.

In addition to host genetics, many other factors play a central role in MVV infection, such as viral dose, route of transmission, management, or coinfection with other diseases (7, 17, 64, 88). Another factor influencing host susceptibility for MVV infection could be a genetic adaptation of the virus to host resistance (25). Clawson et al. (87) reported that a subgroup of the SRLV A2 strain can infect sheep with the *TMEM154 A4Δ53* frameshift mutation, even if this abolishes the protein function and thus prevents infection with other genotypes of the virus. However, the present study could not confirm this finding, as none of the sheep homozygous for A4Δ53 tested positive for MVV. A similar phenomenon was first described by Sider et al. (25) regarding the amino acid exchange at *TMEM154* position 35 in relation to different SRLV genotypes prevalent in the USA. In our study, SRLV genotyping in the four seropositive sheep which carried genotype KK at *TMEM154* codon 35 delivered only a result in two sheep, serotype B by ELISA, and genotype A21 by sequencing, respectively (Table 5). Therefore, no conclusions can be drawn from these results regarding the influence of SRLV genotypes present in Germany on the susceptibility of sheep with the KK genotype at position 35 of *TMEM154.*

In several breeds, frequencies of the *TMEM154* genotypes at position 35 were consistent with the known breed susceptibility (85). In a German study, the highly MVV-susceptible sheep breeds Texel, East Friesian Milk, and Cameroon showed low frequencies of the protective genotype (0–10%), whereas Merinoland and German Grey Heath sheep, not considered to be susceptible to MVV, exhibited 75% and almost 100% of KK genotype, respectively (89). The RPL breed had a KK genotype frequency of 28% and is therefore somewhere between the highly MVV-susceptible and less susceptible German breeds. With the K allele present in 53% of the genotyped RPL sheep, selective breeding would be a realistic opportunity to reduce MVV prevalence, particularly in affected RPL flocks. As the putatively second protective variant, *TMEM154 A4Δ53*, is also present in the RPL breed, it should be considered to enlarge the selection also for sheep carrying the linked allele M at codon 44 of *TMEM154*. This approach has potential advantages and disadvantages. On the one hand, it has to be kept in mind that the biological role of the TMEM154 protein is not fully understood, therefore it is not known if it would be better to keep a full-length protein. On the other hand, a second protective allele with a reasonable allele frequency in the breed (17.6%) would allow to keep more sheep for breeding and thus to better maintain genetic diversity. Moreover, the presence of more than one protective variant may prevent an adaptation of the virus to the host’s defence mechanism.

## 5 Conclusion

This study highlighted for the first time the presence of MVV in the German sheep breed RPL. Although prevalence was low, the disease should not be underestimated, as it can spread unnoticed in the absence of serological monitoring. Therefore, routine serological testing is the most cost-effective preventive strategy, especially in purchased sheep, as the introduction of new ewes with an unknown MV status was identified as main risk factor. Heterogeneous results of the SRLV serotyping and genotyping indicate that MVV does not have a single origin but was introduced into the RPL breed via several different pathways. Moreover, the association between the variation at position 35 in the coding region of *TMEM154* gene and MVV susceptibility tended to be significant and may be influenced by the MVV genotype. Nearly 50% of the genotyped RPL sheep carried protective *TMEM154* genotypes (35KK, 44MM, or 35EK in combination with 44MT), providing a promising foundation for the implementation of selective breeding strategies aimed at reducing MVV susceptibility within this breed. Among the protective alleles, *TMEM154* allele K appears most suitable for breeding programs, as it was more frequent than allele M, encodes a full-length TMEM154 protein, and has shown comparable protective effects in other breeds. However, further investigation is needed to determine whether selective breeding for lower susceptibility could serve as an additional tool to eradicate the disease, both within affected flocks and across the entire breed.

## Data Availability

The datasets presented in this article are not readily available because they originate from German sheep farms and therefore contain privacy issues. Requests to access the datasets should be directed to the corresponding author with the permission of the sheep farmers.
